# Embedded Ceria Nanoparticles in Crosslinked PVA Electrospun Nanofibers as Optical Sensors for Radicals

**DOI:** 10.3390/s16091371

**Published:** 2016-08-26

**Authors:** Nader Shehata, Effat Samir, Soha Gaballah, Aya Hamed, Asmaa Elrasheedy

**Affiliations:** 1 Department of Engineering Mathematics and Physics, Faculty of Engineering, Alexandria University, Alexandria 21544, Egypt; aya_ali@mena.vt.edu; 2 Center of Smart Nanotechnology and Photonics (CSNP), Smart CI Research Center, Alexandria University, Alexandria 21544, Egypt; effat_samir@mena.vt.edu (E.S.); soha_gaballah@mena.vt.edu (S.G.); Asmaa.adel29@gmail.com (A.E.); 3 USTAR Bioinnovations Center, Utah State University, Logan, UT 84341, USA; 4 Department of Electrical Engineering, Faculty of Engineering, Alexandria University, Alexandria 21544, Egypt; 5 Department of Chemical Engineering, Faculty of Engineering, Alexandria University, Alexandria 21544, Egypt

**Keywords:** ceria nanoparticles, electrospinning, crosslinking, fluorescence quenching, radicals

## Abstract

This work presents a new nanocomposite of cerium oxide (ceria) nanoparticles embedded in electrospun PVA nanofibers for optical sensing of radicals in solutions. Our ceria nanoparticles are synthesized to have O-vacancies which are the receptors for the radicals extracted from peroxide in water solution. Ceria nanoparticles are embedded *insitu* in PVA solution and then formed as nanofibers using an electrospinning technique. The formed nanocomposite emits visible fluorescent emissions under 430 nm excitation, due to the active ceria nanoparticles with fluorescent Ce^3+^ ionization states. When the formed nanocomposite is in contact with peroxide solution, the fluorescence emission intensity peak has been found to be reduced with increasing concentration of peroxide or the corresponding radicals through a fluorescence quenching mechanism. The fluorescence intensity peak is found to be reduced to more than 30% of its original value at a peroxide weight concentration up to 27%. This work could be helpful in further applications of radicals sensing using a solid mat through biomedical and environmental monitoring applications.

## 1. Introduction

Radical sensing is important in different applications including cancer treatment [1,2] and water quality monitoring [3]. One source of radicals in experiments is peroxides, due to the unstable oxygen-oxygen chemical bond and consequently the creation of active radicals [4]. Regarding the techniques for sensing radicals, the optical fluorescence-based sensing mechanism is better than other electrochemical ones as it does not require a reference electrode and it is immune to exterior electromagnetic field interference [5,6,7]. Cerium oxide nanoparticles (ceria NPs) can be considered one of the most promising nanostructures which can capture the radicals depending on their formed O-vacancies associated to the active trivalent ionization state of cerium [8,9,10,11]. In this novel work, we aimed to use ceria nanoparticles as an optical sensor for peroxides or the corresponding radicals. However, our ceria NPs would be in the form of a solid host instead of being colloidal solutions so that they could be flexibly used in a wide variety of applications. Therefore, our synthesized nanoparticles are embedded in-situ with polyvinyl alcohol (PVA) nanofibers formed by an electrospinning technique. Then, the formed nanocomposite is used as an optical sensing mat for peroxide or radicals in solution. PVA was selected as a non-toxic biodegradable water soluble polymer [12,13]. Regarding the formation of PVA nanofibers, an electrospinning technique is selected as the fabrication method for the proposed nanocomposite because of the simplicity of operation, the feasibility to embed ceria NPs in the resulting PVA nanofibers, and the potential for scale-up to manufacture large volumes [14,15]. In electrospinning, a high strength electric field is applied between a metallic needle and a metallic target. The strength of the electric field forces the polymer droplets at the needle tip to stretch and to be deposited into fibers on the surface of the target. To make the nanofibers (NFs) suitable for sensing radicals in solution, the formed nanocomposite has been crosslinked via a chemical esterification method, therefore, the produced nanofibers (NFs) can resist solubilization and became a hydrophobic material. Different characterizations of the formed nanocomposite are presented in this work, including absorbance dispersion, bandgap calculation, fluorescence spectroscopy, SEM, and FTIR spectroscopy. The studied optical characterizations prove that the synthesized nanocomposite has some trivalent cerium ions which can be active receptors for radicals adsorption through the associated formed O-vacancies [16]. Then, the fluorescence emissions of the formed nanocomposite in the presence of different concentrations of peroxide in water solution are studied with calculating the Stern-Volmer constant in the linear region of the relative intensity change graph. The visible fluorescence emission is obtained under near-UV excitation in a home-made fluorescence spectroscopy setup. The fluorescence intensity is found to be reduced with increasing radical concentration due to a fluorescence quenching mechanism.

## 2. Materials and Methods

### 2.1. Nanoparticle and Polymer Synthesis

Ceria nanoparticles were synthesized using a chemical precipitation technique, similar to that described in [17,18], as it is a relatively simple procedure using low cost precursors. During ceria preparation, cerium (III) chloride heptahydrate (0.5 g, 99.9%, Sigma-Aldrich, St. Louis, MO, USA) is added to deionized (DI) water (40 mL), then ammonia (1.6 mL) is added as a catalyst. The solution is stirred over a magnetic stirrer for 24 h in an open container at 500 rpm. During the first 2 h of the stirring, the container is held in 50 °C water bath and then the solution is allowed to cool to room temperature for the remainder of the stirring period. The long duration of the stirring helps in fracturing nanorods into nanoparticles. The solution is then centrifuged and washed twice with both deionized water and ethanol to remove any unreacted cerium and ammonia. PVA solution is prepared by mixing PVA pellets(10 g, M_w_ = 205,000 g/mol, Sigma Aldrich, St. Louis, MO, USA) with distilled water to make 100 mL of solution. The solution is heated to 100 °C for 30 min then stirred overnight. Different weight percentages of ceria NPs are added in-situ to the PVA solution. The mixture is stirred for 30 min before it is used in the electrospinning process.

### 2.2. Electrospinning and Crosslinking

The electrospinning setup consists of high voltage power supply (model CZE1000R, Spellman High Voltage Electronics Corporation, Hauppauge, New York, NY, USA), a syringe pump (NE1000-Single Syringe Pump, New Era, Farmingdale, New York, NY, USA) which is used to regulate the feed rate of polymer solution, a 5 mL plastic syringe with an 18 gauge metallic needle to hold the polymer solution, and a circular metallic collector of radius 10 cm covered with aluminum foil is used as a target. The voltage power supply is connected to the needle while the collector is grounded. The distance between the needle tip and the collector is fixed at 15 cm. The voltage difference between the needle and target is 25 kV. The flow rate of the polymer solution is fixed at 2 mL/h. The running time of electrospinning process per sample is about 30 min. A high voltage is applied to the syringe’s needle; the droplet of polymer solution takes the shape of a cone known as Taylor cone [19]. Then, a jet is erupted from the cone and is accelerated towards the collector. As the jet travels through the air, the solvent evaporates leaving behind fibers to be collected randomly on the grounded target. For crosslinking of the formed nanofibers, Vapor phase esterification process was done in the oven on two subsequent steps. In the first step, electrospun nanofibers of PVA or PVA-doped ceria were placed in a container along with a small amount of malic acid (1–2 g) and a few drops of HCl were added to the malic acid. The container was sealed from the ambient moisture and placed in an oven at 80 °C for 15 min. Esterification occurred via a heterogeneous reaction during 15 min. In the second step, the sample was cured for 20 min in an oven at 120 °C.

### 2.3. Nanocomposite Characterizations

PVA nanofibers with embedded ceria NPs are optically characterized by measuring theoptical absorbance, and fluorescence intensity curves. Optical absorbance in the wavelength range from 300 to 700 nm was measured by using a T92+UV-Vis spectrophotometer (PG instruments, Beijing, China). From the absorbance curves, the corresponding NFs band gap can be determined, as explained in the Results and Discussion sections. The mean synthesized nanoparticle size was determination of observed by JEM-2100F transmission electron microscopy (JEOL, Tokyo, Japan) with an accelerating potential of 80 KV. Surface morphology and diameter size of electrospun nanofibers before and after esterification were investigated using scanning electron microscopy (Quanta 200, FEI, Hillsboro, OR, USA). After sputter-coating with gold, the fiber size distribution of randomly selected SEM micrographs was measured using the Image-J software. The formed nanocomposites have been characterized using IR tracer-100 FTIR spectroscopy (Shimadzu, Kyoto, Japan).

### 2.4. Fluorescence Quenching Experiment

**Figure 1:**
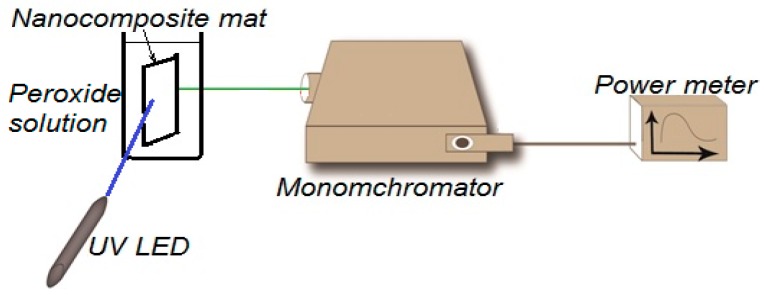
Fluorescence intensity home-made spectroscopy setup.

Fluorescence intensity measurements are done by a home-built fluorescence spectroscopy setup, shown in Figure 1. The fluorescence setup is composed of an ultraviolet (UV) LED with 430 nm excitation wavelength, a Cornerstone 130 Monochromator (Newport, Irvine, CA, USA); which is set to obtain the fluorescence intensity at wavelengths from 500 to 700 nm, an Oriel photomultiplier tube (Newport PMT77340) as a fluorescence intensity detector, and a Newport 1918-R power meter to display PMT detection readings. Fluorescence intensity is measured by positioning the NFs solid sample holder at a 45° between the UV-LED and the input port of the monochromator. The output port of the monochromator was directly connected with the PMT, which was directly connected to the 1918-R power meter.

To test the ability of our synthesized nanocomposite to attract free radicals, hydrogen peroxide solution (35 wt %, Sigma-Aldrich) samples with different volume percent areused. The sample holder with the synthesized nanocomposite mat, a piece of 0.5 cm × 1.5 cm, is inserted in a 100 mL beaker which contains the radicals at different weight concentrations. At each peroxide weight concentration, the fluorescence intensity peak emitted from the contacted nanocomposite with error bars has been detected and monitored by the power meter.

## 3. Results

### 3.1. Nanocomposite Charcterization

**Figure 2:**
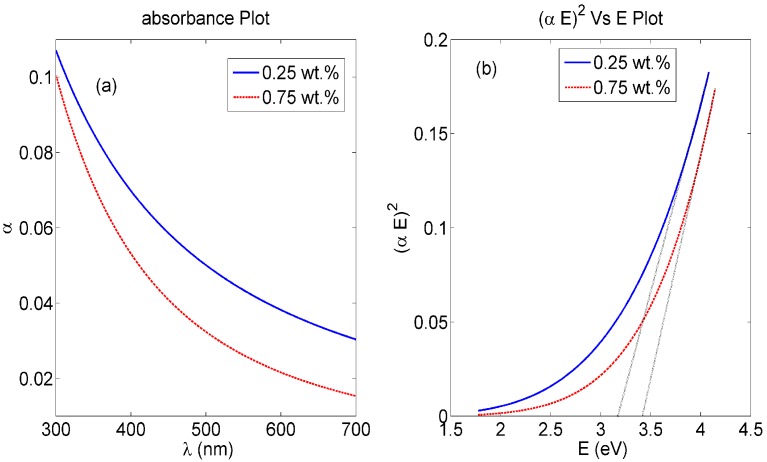
PVA NFs with in-situ embedded ceria NPs. (**a**) Absorbance curve; (**b**) Band gap curve.

The absorbance dispersion curves of our synthesized nanocomposite PVA NFs with embedded ceria NPs with some weight concentrations are shown in Figure 2a. The corresponding direct bandgap can be calculated from the absorbance dispersion curve through Equation (1) [20]:

(1) $$\alpha (E)=A{\left(E-E_{g}\right)}^{1/2}$$

where α is the absorbance, *A* is a material-dependent constant which includes both the effective masses of electrons and holes and dielectric constant of the nanocomposite, *E* is the absorbed photon energy (*E* = *hc*/λ) as λ is the optical absorbed wavelength, and *Eg* is the allowed direct bandgap of the synthesized nanocomposite. Then, the relation between (αE)^2^ and E is presented in Figure 2b. The intersection of the extrapolation of the linear part of (αE)^2^ curve with E-axis is equal to allowed direct bandgap *Eg*, that particular composition of ceria NPs with moderate concentration of tri-valent cerium ionization states with associated O-vacancies, which is within accepted range biased to 3 eV [16,21].

**Figure 3:**
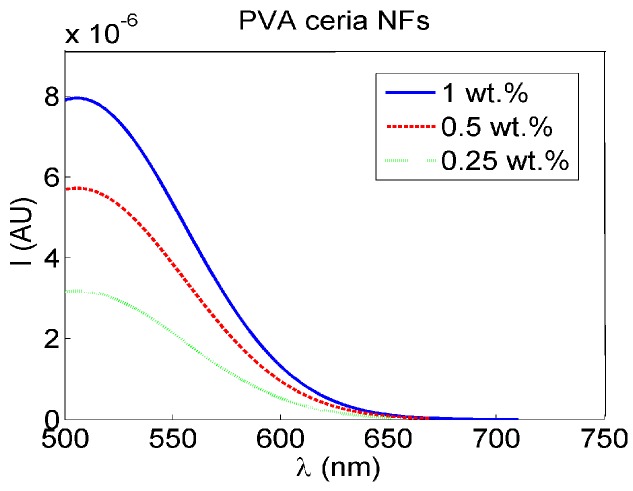
Fluorescence intensity PVA NFs with in-situ embedded different concentrations ceria NPs.

Visible fluorescence intensity spectra, under 430 nm optical excitation, are shown in Figure 3 for different concentrations of ceria NPs which are embedded in-situ in crosslinked PVA NFs. Generally, our synthesized nanocomposite mat displays the same optical activity properties as ceria nanoparticles in a biodegradable solid and flexible host of PVA nanofibers for a wide variety of possible applications.

**Figure 4:**
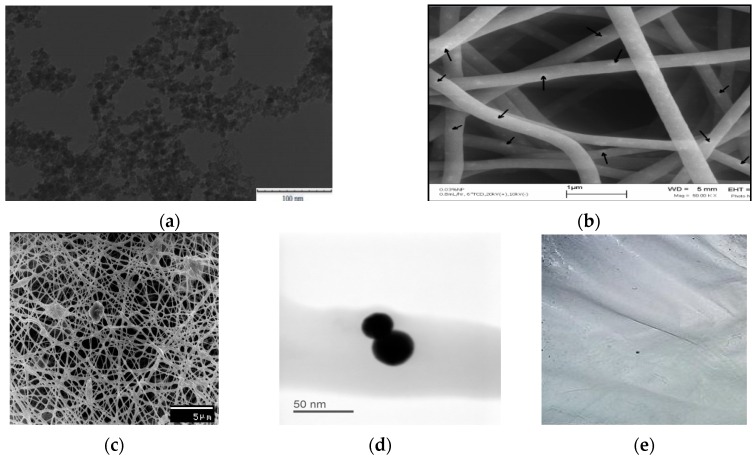
(**a**) TEM image of ceria NPs; (**b**) SEM image of our synthesized nanocomposite of PVA nanofibers embedded with ceria NPs; (**c**) SEM image of the nanocomposite after crosslinking; (**d**) STEM image of some agglomerated nanoparticles inside the fiber; and (**e**) macroscopic photo of the synthesized electrospun nanofibers.

**Figure 5:**
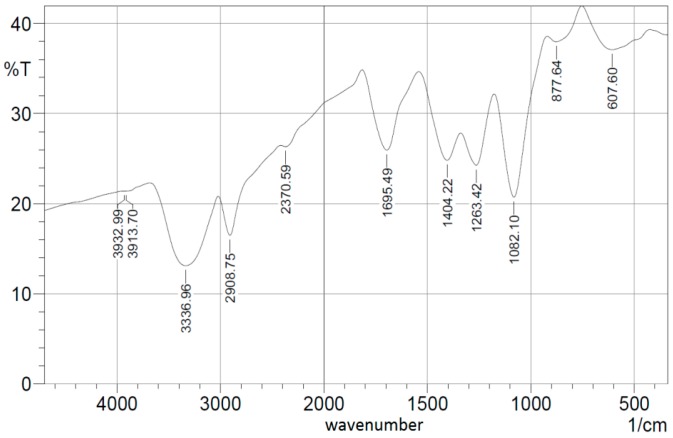
FTIR spectroscopy pattern of crosslinked PVA with embedded ceria NPs.

Figure 4a shows both TEM of ceria NPs with mean diameter less than 10 nm. SEM images of both PVA nanofibers with embedded ceria NPs before and after crosslinking are shown in both Figure 4b,c, with a focused image of some agglomerated nanoparticles inside the fiber shown in Figure 4d. The synthesized nanofibers, in both cases, have mean diameters around 130 nm but with higher numbers of formed beads in the crosslinked case. Some ceria NPs are expected to be located on the surface of the electrospun nanofibers, especially before the crosslinking effect. In addition, a macroscopic photo of the formed crosslinked nanofibers is shown in Figure 4e to show that the formed nanofibers mat is uniform with some minor defects on the sides. Figure 5 shows the FTIR spectroscopy pattern of the crosslinked nanofibers with embedded ceria NPs.

### 3.2. Optical Sensing of Peroxide Using Fluorescence Quenching Mechanism

**Figure 6:**
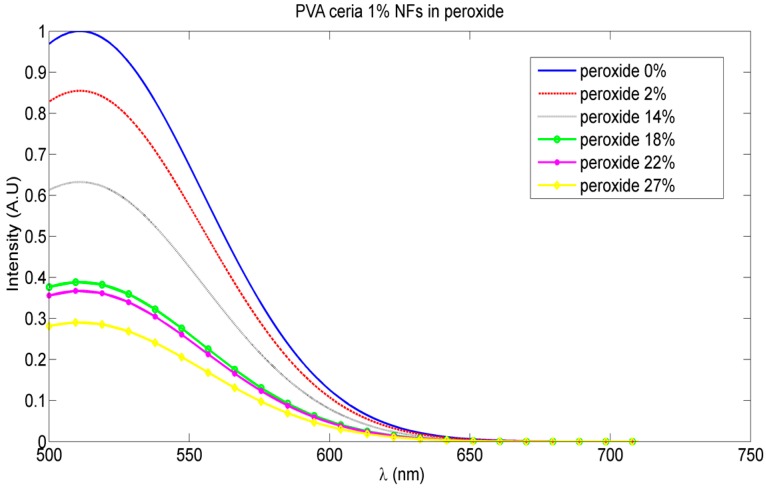
Fluorescence intensity peak degradation with increased peroxide weight concentrations (normalized to the fluorescence intensity peak of the synthesized nanocomposite in the presence of peroxide).

**Figure 7:**
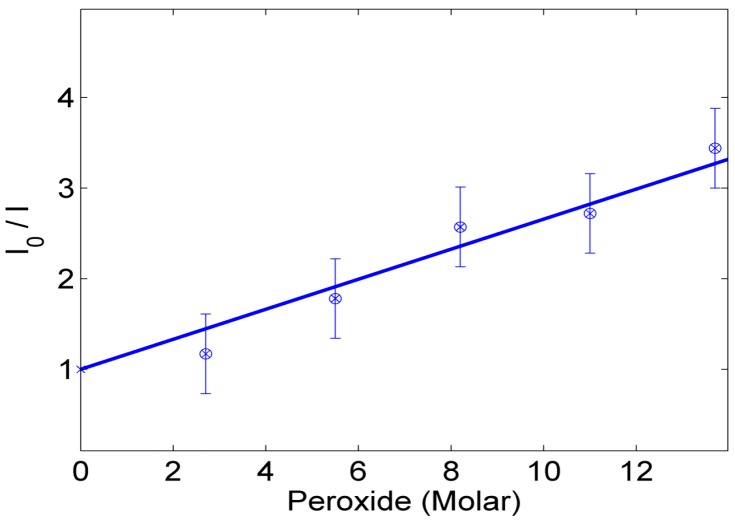
Relative intensity change, compared to the fluorescence intensity peak of nanocomposite in absence of peroxide, versus the variable molar concentrations of peroxide.

The variation of the fluorescence intensity peaks emitted from our synthesized nanocomposite at different peroxide weight concentrations is shown in Figure 6. This proves the fluorescence quenching mechanism is due to the radical quenching sensed by the active ceria NPs embedded in the crosslinked nanofibers. The relative change of fluorescence intensity as a function of the molar concentration of peroxide in water solution is presented in Figure 7.

## 4. Discussion

The bandgap values found from Figure 2 shows evidence for the fluorescence spectra shown in Figure 3, which proves the formation of a visible emission peak at 520 nm under near-UV excitation which confirms the relaxation via the 5d-4f transition of excited Ce^3+^ ions in Ce_2_O_3_, which results in a photon emission [17]. Therefore, the higher concentration of Ce^3+^ states in non-stoichiometric CeO_x_ along with an increase in the associated O-vacancies results in a stronger fluorescence emission [16]. The authors tried higher weight concentrations of embedded ceria NPs in the host material, but the fluorescence intensity peak becomes lower, because the static quenching of higher nanoparticle concentrations becomes dominant. In addition, the higher concentrations of ceria nanoparticles lead to some droplets in the nanofiber mat due to lower viscosity. That leads us to conclude that 1 wt % may be the optimum for our current studied nanocomposite and its targeted application. In the FTIR spectrum shown in Figure 5, most of the original peaks of both PVA and malic acid, including OH alcohol, free hydroxyl, C-H alkane, C-O carboxylic acid, ester and C=O carboxylic bonds, are not affected through embedding ceria NPs. Supported by Figure 4b, the FTIR analysis is important to show that ceria nanoparticles with their corresponding vacancies may have higher possibility to be on the surface of the nanofibers with better sensitivity to adsorb and sense the radicals in the aqueous medium.

Figure 6 shows the reduction of the fluorescence intensity emitted from our synthesized nanocomposite more than 30% with increase of radicals or peroxide weight concentration up to 27%. The relation between the amplitude of the fluorescence signal and the quencher concentration; the peroxide or the corresponding radicals, is clarified in Figure 7. The linear part is described by the Stern-Volmer equation, as follows [22]:

(2) $$\frac{I_{o}}{I}=1+K_{SV}\left[Q\right]$$

where *I_o_* and *I* represent the peak intensities of the steady-state fluorescence in the absence and presence, respectively, of the peroxide quencher, *K_SV_* is the Stern-Volmer quenching constant which is an indication for the sensitivity of the nanocomposite to sense the radicals [7], and [Q] is the quencher concentration. The results of the analysis of the fluorescence data as functions of radicals concentration are shown in Figure 7. The *K_SV_* value of the synthesized nanoparticles is calculated from the slope of the fitted line, and found to be 0.037 M^−1^.

## 5. Conclusions

This work introduces a novel nanocomposite of ceria NPs embedded in crosslinked PVA electrospun nanofibersas an optical sensing mat for peroxide and the corresponding radicals. Our synthesized nanocomposite proves the activity of the embedded ceria NPs to have some Ce^3+^ ionization states and the associated charged O-vacancies to be receptors for radical adsorption. In addition, the synthesized nanocomposite is found to be fluorescent with visible emission under near-UV excitation. The visible fluorescence intensity peak is found to be reduced with increasing peroxide concentration through a fluorescence quenching mechanism. Through optical fluorescence, the intensity could be reduced clearly up to 30% of its original value in the absence of radicals with peroxide concentrations up to 27 wt %. This study could be extensively helpful in further applications in environmental monitoring and cancer detection.

## Abbreviations

The following abbreviations are used in this manuscript:

Ceria NPs	Cerium oxide nanoparticles
PVA	Polyvinyl alcohol
SEM	Scanning Electron Microscope
TEM	Transmission Electron Microscope
